# Synergistic Insecticidal Efficacy of the Natural Fumigant Methyl Benzoate Against Phosphine-Resistant *Sitophilus oryzae*

**DOI:** 10.3390/life16101688

**Published:** 2026-10-09

**Authors:** Hwal-Su Hwang, Mst. Fatema Khatun, Md Munir Mostafiz, Jun-Ran Kim, Bong-Su Kim, Soo-Jung Suh, Kyeong-Yeoll Lee

**Affiliations:** 1 Department of Plant Medicine, Kyungpook National University, Daegu 41566, Republic of Korea; bgtwo2@naver.com; 2 Department of Entomology, Gazipur Agricultural University, Salna, Gazipur 1706, Bangladesh; fatema20@gau.edu.bd; 3 Crop Science Department, Teagasc, Oak Park, R93XE12 Carlow, Ireland; munirmostafiz12@gmail.com; 4 Plant Quarantine Technology Center, Animal and Plant Quarantine Agency, Gimcheon 39660, Republic of Korea; junrankim@korea.kr (J.-R.K.); bskim79@korea.kr (B.-S.K.); suhsj97@korea.kr (S.-J.S.); 5 Institute of Plant Medicine, Kyungpook National University, Daegu 41566, Republic of Korea

**Keywords:** rice weevil, methyl benzoate, phosphine resistance, co-fumigation, Bliss synergy, dihydrolipoamide dehydrogenase

## Abstract

The widespread emergence of phosphine-resistant populations of stored-product pests, such as the rice weevil (*Sitophilus oryzae*), poses a significant challenge to effective grain protection. This study investigated the fumigation toxicity of methyl benzoate (MBe), an eco-friendly aromatic ester, both alone and in combination with phosphine against phosphine-susceptible (So-PS) and phosphine-resistant (So-PR) strains of *S. oryzae*. Molecular analysis identified an asparagine-to-threonine substitution in the dihydrolipoamide dehydrogenase (*dld*) gene in the So-PR strain, which exhibited a 141-fold higher resistance to phosphine than the So-PS strain of LC_50_ = 1.13 and 0.008 mg/L, respectively. In contrast, MBe exhibited negative cross-resistance, with greater toxicity toward the So-PR strain (LC_50_ = 6.10 mg/L) than toward the So-PS strain (LC_50_ = 10.08 mg/L). Co-fumigation assays based on a Bliss independence model revealed strong synergistic fumigation toxicity across the tested concentration matrix. Combining MBe at the strain-specific LC_30_ with reduced phosphine concentrations resulted in complete mortality of So-PR adults under the tested conditions. The observed synergy is consistent with the involvement of distinct toxicological targets of the two fumigants, although this study did not directly examine the underlying mechanism. These findings indicate that MBe has potential as a co-fumigant for managing phosphine-resistant *S. oryzae* and warrants further evaluation under grain-bulk and storage conditions.

## 1. Introduction

With advances in agricultural technology, crop production has increased, leading to the expansion of storage and international trade of harvested agricultural commodities. Losses of stored grain vary widely with storage conditions and management. Insect pests are among the principal causes of quantitative and qualitative deterioration, with losses commonly reported at 10–30% under traditional tropical storage conditions and reaching 50% or more in severely infested or poorly managed storage systems [1,2,3]. To date, more than 1660 insect pest species have been reported to cause problems during storage, of which approximately 70 are recognized as major stored-product pests responsible for substantial economic losses [4]. Among these, the rice weevil, *Sitophilus oryzae* (Coleoptera: Curculionidae), is a major pest of stored grains. Both adults and larvae continuously infest not only rice but also wheat, corn, barley, and sorghum, causing weight loss, quality deterioration, putrefaction, and fungal growth, thereby posing a serious threat to food safety [5].

Unlike many other pests that feed on the surface of grains, *S. oryzae* females have the distinctive ability to oviposit inside grain kernels, where the larvae subsequently develop [6,7]. Because of this internal feeding behavior, control with conventional contact insecticides is extremely difficult, requiring the use of gaseous fumigants capable of penetrating deep into grain kernels [8]. To control stored-product pests that cause both internal and external damage, methyl bromide (MB) was widely used as a primary fumigant in Korea until the early 2000s. However, its widespread use has since been banned because of concerns regarding ozone layer depletion and human toxicity [9]. Phosphine was subsequently introduced as an alternative and remains widely used. However, the emergence of phosphine-resistant pest populations has increased the need to develop novel fumigants [10]. Molecular studies have revealed that phosphine resistance across multiple pest species is often associated with highly conserved point mutations in the dihydrolipoamide dehydrogenase (*dld*) gene, highlighting the need for fumigants with distinct modes of action [11]. Consequently, other alternative chemical fumigants, such as ethyl formate (EF) and carbonyl sulfide (COS), are used but they present challenges, including flammability, heavy sorption by grain, and the need for high application doses [12], as well as toxicological and residue concerns [13].

As a defense mechanism against external threats, plants produce secondary metabolites, many of which have attracted attention as eco-friendly pest control agents [14,15]. Among these natural compounds, terpinen-4-ol, linalool, isopinocamphone, and methyl benzoate (MBe) are eco-friendly and have low human toxicity, allowing approval as food additives. In particular, MBe is an aromatic ester naturally produced by various plants and exhibits high insecticidal activity against several agricultural and stored-product pests, including the lesser grain borer, Indianmeal moth, and rice weevil [16,17,18]. Furthermore, MBe has little effect on pollinators such as bees or natural enemies such as parasitoid wasps, making it a promising green fumigant for selective pest control [19,20].

Recent studies have shown that MBe acts on the insect nervous system by significantly inhibiting acetylcholinesterase (AChE) activity in a variety of insect species, including the cotton aphid, *Aphis gossypii* (Hemiptera: Aphididae), the bean weevil, *Acanthoscelides obtectus* (Coleoptera: Chrysomelidae), and the house fly, *Musca domestica* (Diptera: Muscidae) [21,22,23]. Phosphine and MBe have different reported toxicological effects, with phosphine primarily affecting cellular respiration and MBe potentially affecting the nervous system [22,24]. These differences provide a rationale for investigating their combined effects, although the mechanism underlying any interaction must be established experimentally. These differences provide a rationale for investigating their combined effects, although the mechanism underlying any interaction must be established experimentally.

Therefore, the objective of this study was to evaluate the fumigant toxicities of MBe and phosphine, individually and in combination, against phosphine-susceptible (So-PS) and phosphine-resistant (So-PR) strains of *S. oryzae*, assess the differential susceptibility of the two strains to MBe as an indicator of potential negative cross-resistance, and characterize the interactions between MBe and phosphine using the Bliss independence model.

## 2. Materials and Methods

### 2.1. Rearing of Sitophilus oryzae

**Figure 1:**
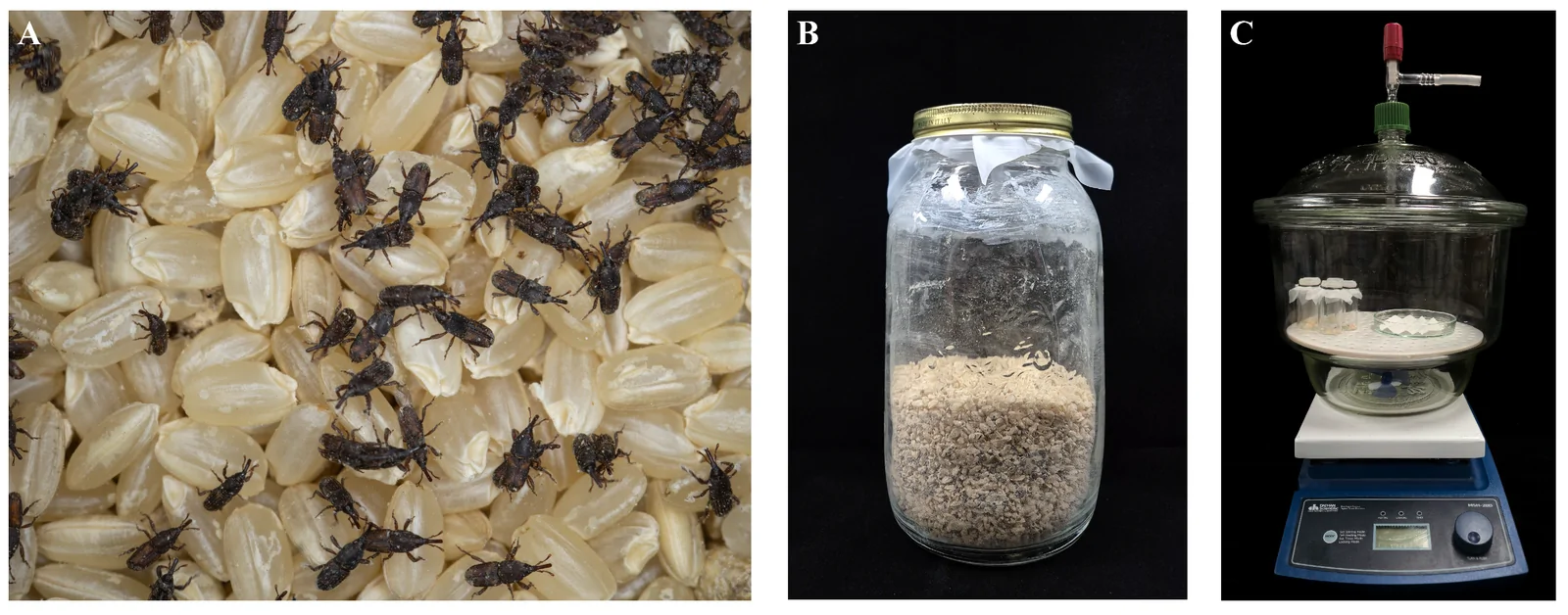
Rearing and fumigation procedures for *S. oryzae*. Mass rearing of *S. oryzae* in brown rice (**A**) in the rearing jar (**B**) and the glass desiccator used for fumigation experiments, equipped with an internal air-circulation device consisting of a magnetic stirrer and propeller (**C**).

The phosphine-susceptible (PS) and phosphine-resistant (PR) strains of the rice weevil, *S. oryzae*, used in this study were obtained from the Plant Quarantine Technology Center of the Animal and Plant Quarantine Agency (Gimcheon, Republic of Korea). The PR strain consisted of a population with a history of survival at the phosphine-discriminating concentration, whereas the PS strain served as the susceptible control. Both strains were reared in an incubator maintained at 25 ± 1 °C and 60 ± 5% relative humidity under a 14:10 h light:dark (L:D) photoperiod. Glass jars with a capacity of 1.7 L were used for insect rearing, and 500 g of intact brown rice (or white rice) was provided as the rearing medium to allow female *S. oryzae* to oviposit inside the grain kernels. To prevent moisture accumulation inside the jars and minimize insect escape, an approximately 5-cm-diameter hole was made in the center of each 8.6-cm-diameter lid and covered with a 150-mesh net (Figure 1A,B). Furthermore, to prevent adult *S. oryzae* from crawling toward the lid, a polytetrafluoroethylene solution was applied to the upper inner wall of the jar opening. The net covering the lid provided an additional barrier against escape.

Adults were separated from the rice every 7 days using a 13-mesh sieve and transferred to fresh rice in new glass jars to maintain continuous oviposition and reproduction. The separated rice, which contained *S. oryzae* eggs and larvae developing inside the kernels, was stored separately under identical rearing conditions. Newly emerged adults were subsequently collected and used in the experiments. To prevent mite infestation and other external contamination, we sterilized all rice and rearing equipment by heat treatment at 50 °C for 24 h before use. The two strains were subjected to the experiments after healthy colonies had been established through successive laboratory rearing under the conditions described above.

### 2.2. Chemicals

MBe (purity ≥ 99%) was purchased from Sigma-Aldrich (St. Louis, MO, USA). Phosphine was supplied as a 2% mixture of phosphine in CO_2_ (Farm Hannong, Seoul, Republic of Korea).

### 2.3. Analysis of Phosphine Resistance

Before the fumigation experiments, the resistance status of the PS and PR strains of *S. oryzae* was verified using molecular diagnostic methods and bioassays. DNA was extracted from adult *S. oryzae* (*n*= 1 per extraction, with three replicates) of the PR and PS strains using a PureLink™ Genomic Mini DNA Kit (Thermo Fisher Scientific, Wantham, MA, USA) [25]. Polymerase chain reaction (PCR) was performed to amplify a specific region of the dihydrolipoamide dehydrogenase (*dld*) gene, which is associated with phosphine resistance in stored-grain pests [24]. The target region of the *dld* gene was amplified using the primers So-DLDm-F (5′-AGG AGT ACG GCG CAT CAT-3′) and So-DLDm-R (5′-CGA TAA CAA AAA AGG GGC G-3′) [26]. The PCR was performed in a total volume of 30 μL containing 15 μL of Solg™ 2 × Taq Pre-Mix (Solgent, Daejeon, Republic of Korea), 2 μL of each primer (10 pmol/μL), and 3 μL of DNA solution. Sterile double-distilled water (DNase-free water) was then added to obtain a final volume of 30 μL. The PCR conditions consisted of initial denaturation at 92 °C for 5 min, followed by 35 cycles of denaturation at 92 °C for 60 s, annealing at 56 °C for 60 s, and extension at 72 °C for 60 s, with a final extension at 72 °C for 5 min. The amplified PCR products were confirmed via 1% agarose gel electrophoresis and submitted to Solgent for nucleotide sequencing. In parallel, a discriminating-dose bioassay was conducted according to the Food and Agriculture Organization (FAO) guidelines to reconfirm the resistance status of each strain. Adults of each *S. oryzae* strain were exposed to phosphine at a concentration of 0.04 mg/L for 20 h in a sealed desiccator [27].

### 2.4. Fumigation Experiments

All fumigation experiments were conducted in 10-L glass desiccators used as sealed fumigation chambers at 25 ± 1 °C (Figure 1C). Each strain consisted of five replicates, with each replicate consisting of 20 adult *S. oryzae* placed inside a 20-mL mesh-covered glass vial containing 10 grains of brown rice as a minimal food source. To prevent insect escape, the vial was covered with a 150-mesh net instead of a lid, and the net was securely fastened with an O-ring (inner diameter, 16.8 mm; thickness, 2.4 mm). To ensure hermetic sealing, the lid of each 10-L desiccator was sealed with vacuum grease (Dow Enterprise, Asan, Republic of Korea).

Three types of fumigation experiments were conducted: MBe alone, phosphine alone, and co-fumigation of MBe and phosphine. For MBe alone treatment, liquid MBe was applied to No. 10 filter paper (Hyundai Micro, Anseong, Republic of Korea) suspended inside the chamber to allow complete evaporation across a concentration range of 0–16 mg/L. For phosphine alone treatment, gastight syringes were used to inject phosphine (2% in CO_2_) through a top-lid vacuum stopcock (Duran, Wertheim, Germany) to achieve concentrations of 0–2 mg/L. For the co-fumigation treatment, MBe was applied immediately prior to phosphine injection (<1 min gap) across a concentration matrix of 0–8 mg/L MBe (<LC_80_) and 0–0.5 mg/L phosphine (<LC_10_).

Continuous gas circulation was maintained using a magnetic stirrer (Daihan Scientific, Wonju, Republic of Korea) driving a bottom-mounted propeller (Figure 1C). After a 24-h exposure period, chambers were ventilated under a fume hood for 1 h. Adult mortality was assessed under a stereomicroscope (Olympus, Tokyo, Japan) by prodding insects with a fine brush; unmoving insects were recorded as dead. Control groups without fumigant exposure were included in every trial; trials with control mortality > 10% were discarded and repeated.

After 24 h of exposure, the desiccator lid was opened inside a fume hood, and the chamber was ventilated for more than 1 h before the insects were retrieved for mortality assessment. Insecticidal efficacy was evaluated by gently prodding each insect with a fine brush under a microscope (Olympus, Tokyo, Japan) and observing its movement. Insects that showed no response or movement were recorded as dead. All treatments were conducted at 25 ± 1 °C for 24 h.

All experimental procedures were performed inside a chemical fume hood. To ensure operator safety during handling, appropriate personal protective equipment, including nitrile gloves and half-mask respirators equipped with ABEK-P3 cartridges, was worn throughout the experiments.

### 2.5. Statistical and Synergistic Effect Analysis

Mortality data were analyzed to estimate lethal concentrations and compare treatment effects. Dose–response data for each individual fumigant (MBe or phosphine) and strain were subjected to log-probit regression analysis using the PROC PROBIT procedure in SAS 9.4 (SAS Institute, Cary, NC, USA) to estimate LC_30_, LC_50_, LC_90_, and LC_99_ values with their corresponding 95% confidence intervals (95% CIs). The goodness of fit of the model was evaluated using Pearson’s chi-square test.

To assess the synergistic effect of MBe co-fumigation with phosphine against the phosphine-resistant (PR) strain of *S. oryzae*, mortality data were expressed as the mean ± standard error (SE), and statistical analyses were performed using SPSS version 29 (IBM, NY, USA). The normality assumption was assessed using the Shapiro–Wilk test [28], whereas Levene’s test indicated heteroscedasticity [29]. Accordingly, one-way analysis of variance (ANOVA) was used to evaluate overall treatment effects, followed by the Games–Howell post hoc test to determine significant differences among treatments (*p* < 0.05) [30].

The synergistic effects of MBe and phosphine co-fumigation were further evaluated using the Bliss independence model. In this model, *a* and *b* represent the mortality proportions resulting from phosphine and MBe treatments alone, respectively, whereas *O* represents the observed mortality of the combined treatment. The expected mortality under Bliss independence was calculated as *E_Bliss_* = *a* + *b* − *a* × *b*, and the degree of synergy (Bliss excess) was calculated as Δ = *O* − *E_Bliss_* (Δ% = 100 × Δ). A heatmap of Bliss synergy across the combined treatments was generated using R version 4.5.1.

## 3. Results

### 3.1. Molecular Diagnosis of Phosphine Resistance

**Figure 2:**
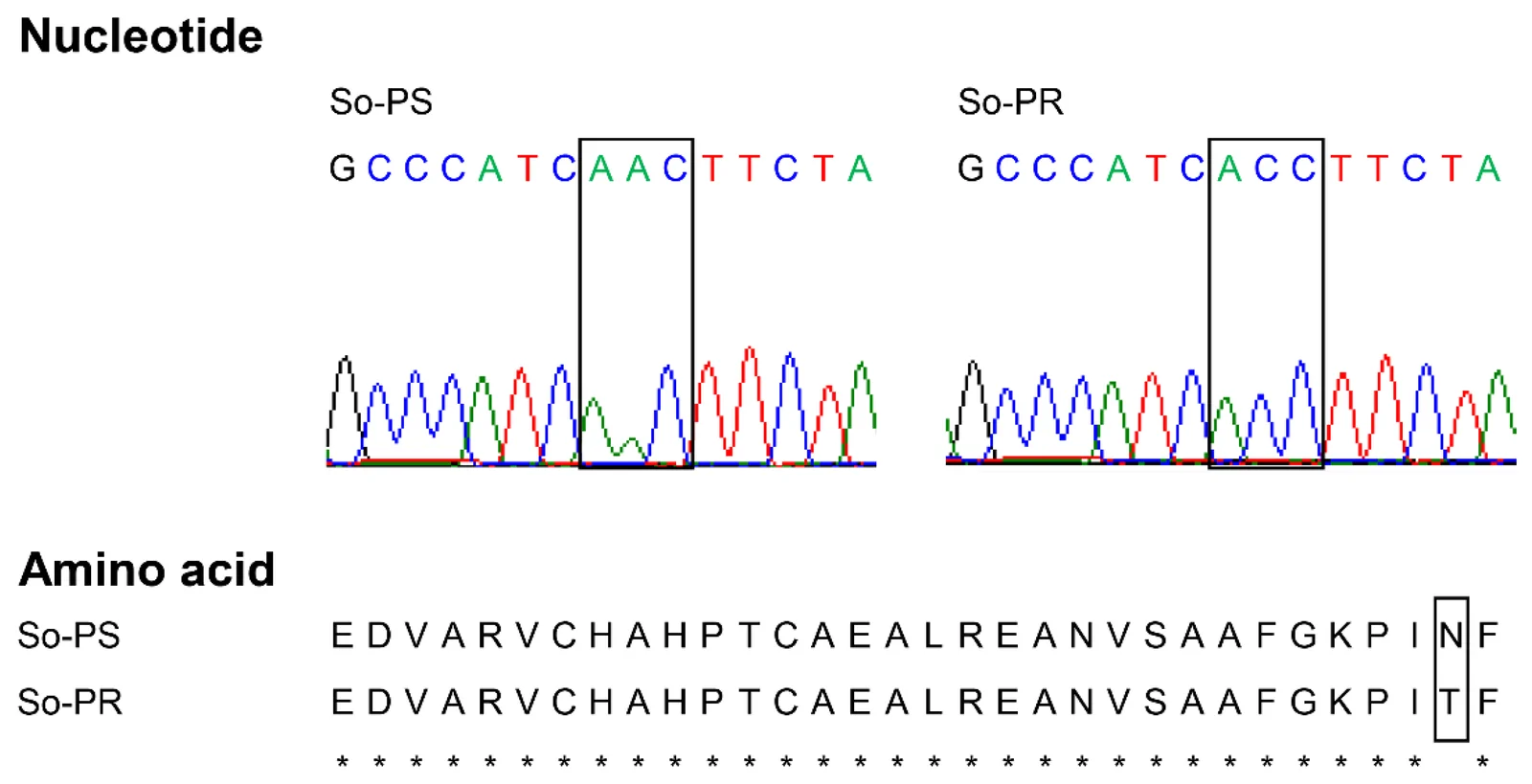
Nucleotide and amino acid sequences of the dihydrolipoamide dehydrogenase (*dld*) gene from *S. oryzae*. In the nucleotide sequence, the colors represent standard chromatogram base color-coding (e.g., A = green, C = blue, G = black, T = red), and black boxes indicate regions that have been translated into amino acid sequences. “So-PS” refers to individuals from the phosphine-sensitive strain, whereas “So-PR” refers to individuals from the phosphine-resistant strain. The two sequences were aligned using Clustal Omega multiple sequence alignment (MSA) version 1.2.4. Asterisks indicate fully conserved amino acid residues between the two strains.

Molecular diagnostic analysis revealed sequence variation in the *dld* gene between the PS and PR strains of *S. oryzae*. Nucleotide sequencing identified a point mutation in which the AAC codon in the susceptible strain was replaced by ACC in the resistant strain. Amino acid sequence alignment confirmed that this nucleotide substitution resulted in the replacement of asparagine (N) with threonine (T) in the resistant strain (Figure 2).

### 3.2. Fumigation Toxicity of Phosphine and MBe

**Figure 3:**
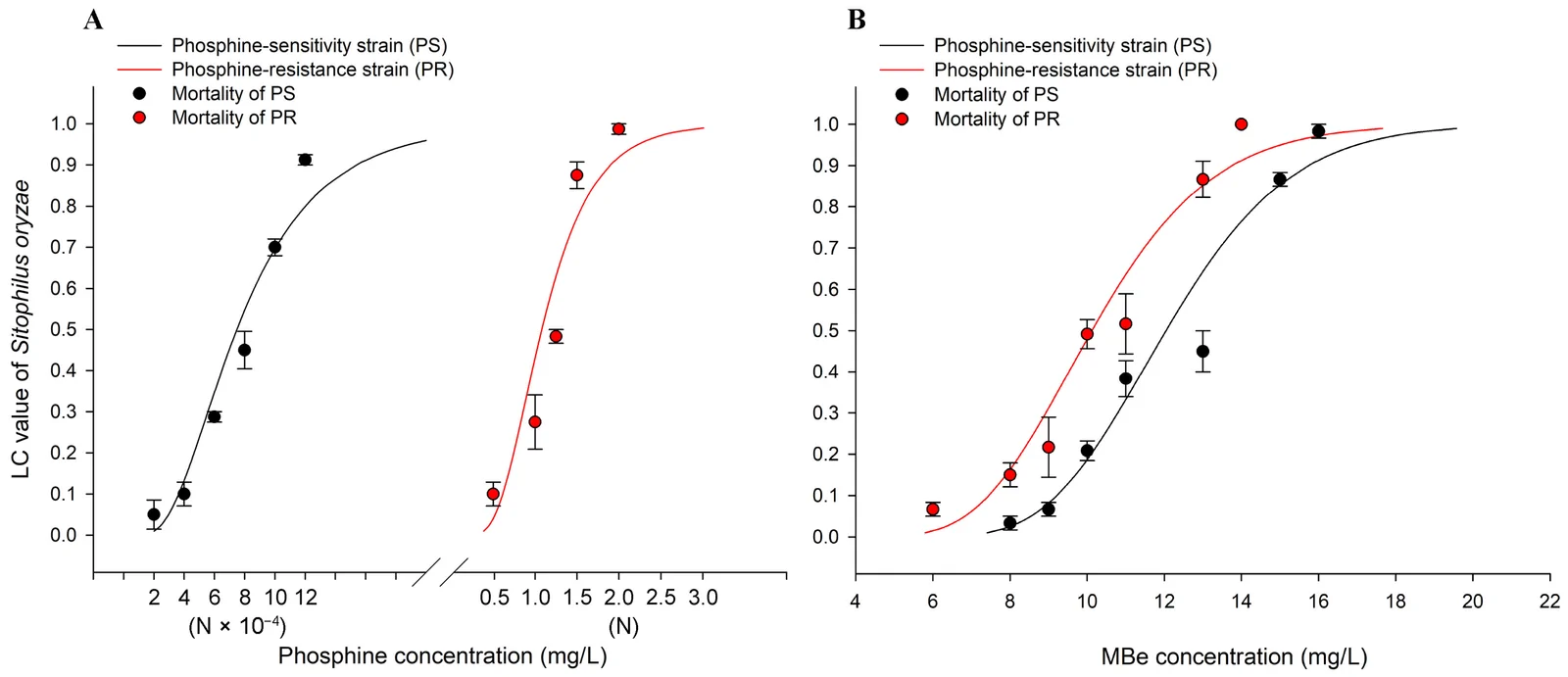
Concentration–mortality responses to phosphine and MBe in the PS and PR strains of *S. oryzae*. Adults (*n* = 20) of the PS strain (black) and PR strain (red) were exposed to various concentrations of phosphine (**A**) or MBe (**B**) for 24 h, and mortality was assessed after 24 h of treatment. Each treatment was performed with five replicates.

Fumigation bioassays evaluating the independent toxicity of phosphine and MBe against *S. oryzae* revealed contrasting toxicity profiles between the susceptible (So-PS) and resistant (So-PR) strains (Figure 3). Phosphine exhibited high toxicity toward So-PS (LC_30_ = 0.006 mg/L; LC_99_ = 0.022 mg/L) but significantly reduced efficacy against So-PR (LC_30_ = 0.85 mg/L; LC_99_ = 3.98 mg/L), corresponding to a 141-fold resistance ratio (Table 1). Notably, MBe exhibited negative cross-resistance, with the So-PR strain showing greater susceptibility than the So-PS strain, as indicated by the lower LC_30_ (5.14 vs. 9.09 mg/L) and LC_99_ (12.98 vs. 15.98 mg/L) values (Table 1).

### 3.3. Synergistic Effects of Combined Fumigation

**Figure 4:**
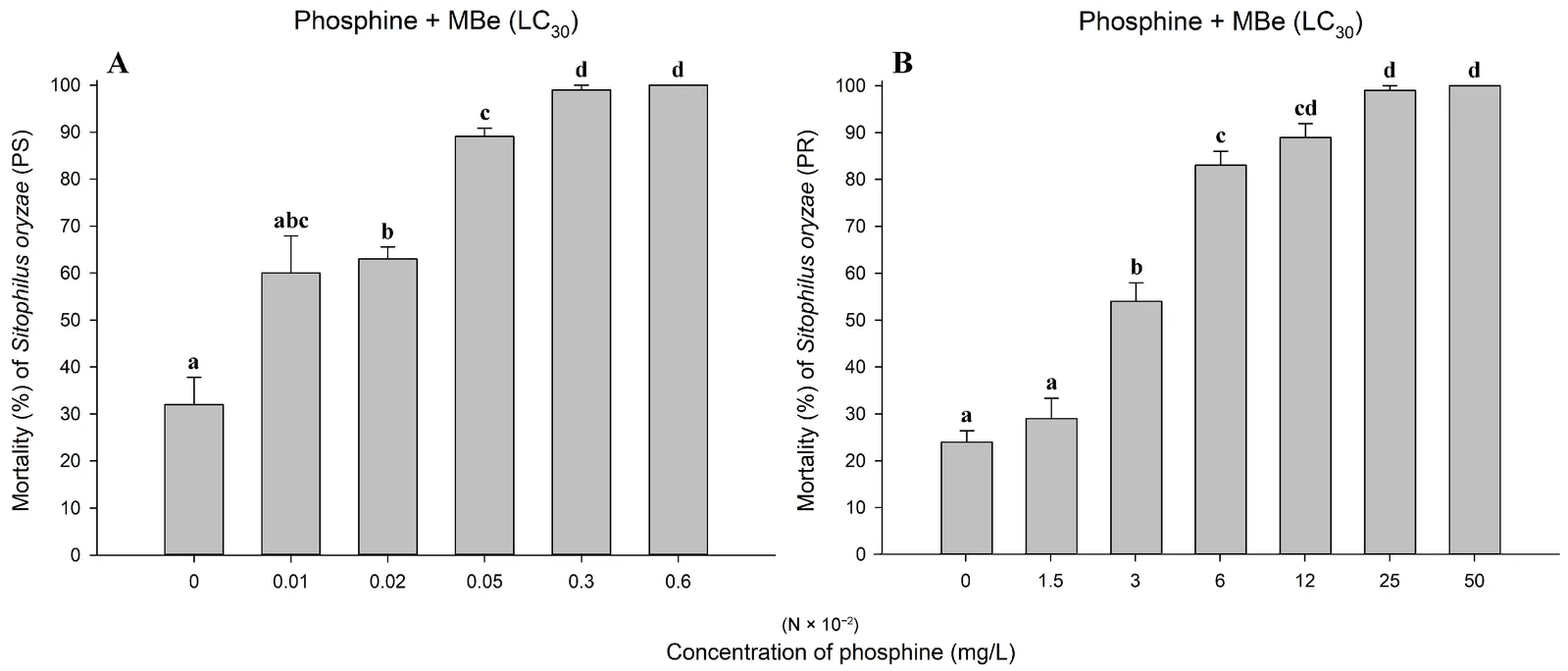
Effects of combined fumigation with MBe and phosphine on adults of the PS and PR strains of *S. oryzae*. The MBe applied at the strain-specific LC_30_ values: 9.09 mg/L for the PS strain and 5.14 mg/L for the PR strain. Phosphine concentrations ranged from 0 to 0.6 × 10^−2^ mg/L for the PS strain (**A**) and from 0 to 0.5 mg/L for the PR strain (**B**). Bars represent mean mortality (%) ± SE. Each treatment was performed with five replicates. Bars with different lowercase letters are significantly different according to the Games–Howell post hoc test (*p* < 0.05).

**Figure 5:**
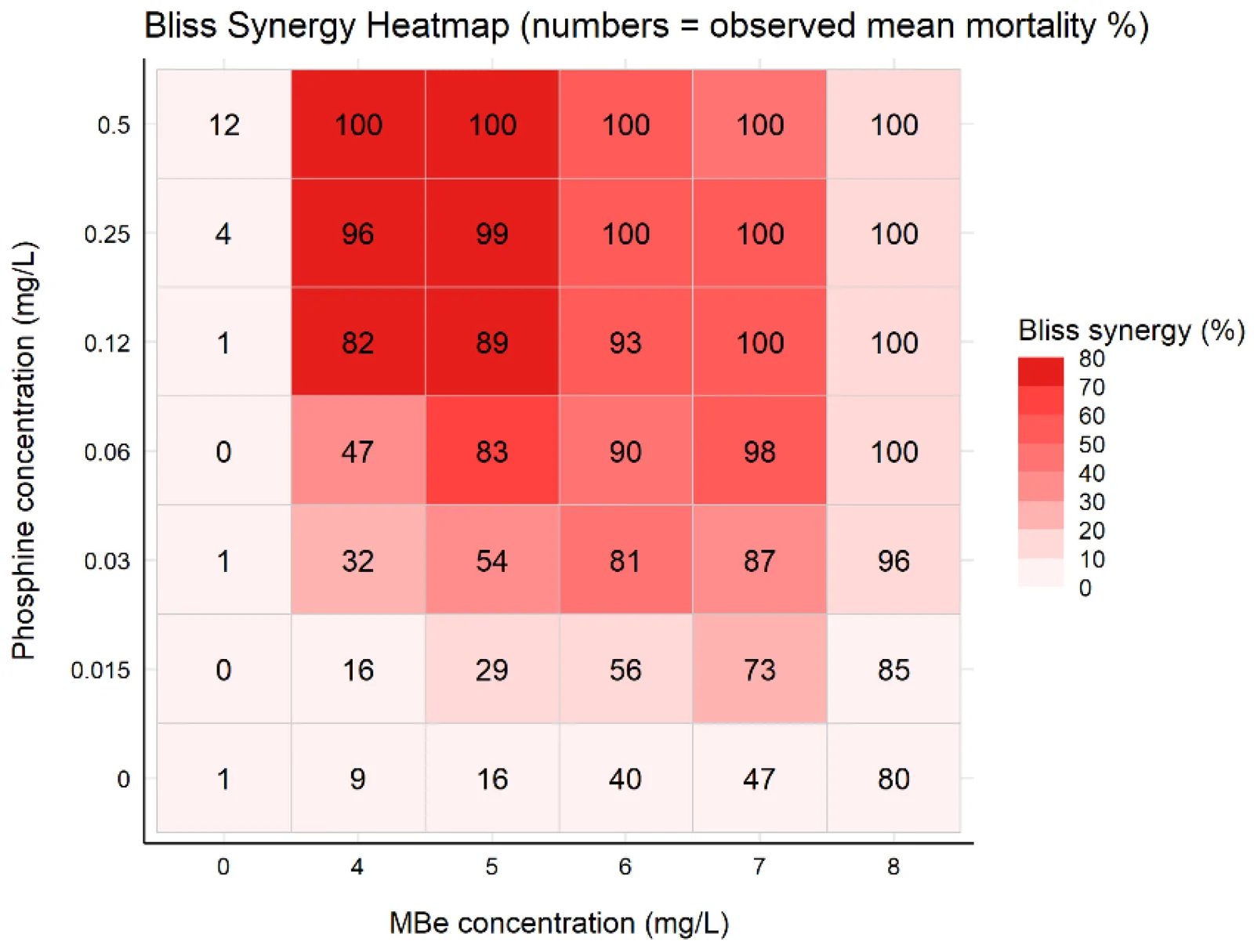
Bliss synergy heatmap for phosphine–methyl benzoate combinations. Cell color represents the Bliss synergy value (red indicates positive synergy). Numbers within the cells indicate the observed mortality (%) of the PR strain of *S. oryzae* at each phosphine–MBe concentration combination (observed mean mortality, %). For interpretation of the references to color in this figure legend, the reader is referred to the Web version of this article.

To evaluate potential synergistic interactions during co-fumigation, MBe was fixed at the strain-specific LC_30_, while phosphine concentrations were systematically varied (Figure 4). In the So-PS strain, increasing the phosphine concentration from 0 to 0.006 mg/L in the presence of a fixed MBe concentration resulted in a rapid increase in mortality, reaching 100%. Remarkably, co-treatment of the resistant strain with MBe at its LC_30_ and 0.24 mg/L phosphine also resulted in nearly 100% mortality, demonstrating that a low concentration of MBe substantially reduced the apparent resistance to phosphine. To further characterize this interaction in resistant *S. oryzae*, MBe concentrations ranging from the LC_10_ (4 mg/L) to approximately the LC_80_ (8 mg/L) were combined with phosphine at concentrations ranging from 0 to 0.5 mg/L, and the resulting interactions were visualized using a Bliss synergy heatmap (Figure 5). The combination of 4 mg/L MBe and 0.12 mg/L phosphine produced 82% mortality, despite the individual treatments producing minimal mortality (9% and <1%, respectively). Complete mortality (100%) was achieved with 4 mg/L MBe + 0.5 mg/L phosphine and with 6 mg/L MBe + phosphine concentrations above 0.25 mg/L. The extensive red regions in the heatmap indicate strong synergistic interactions across a broad range of fumigant concentrations.

## 4. Discussion

This study evaluated the individual and combined fumigation efficacies of MBe and phosphine against *S. oryzae*, a globally important stored-grain pest that causes substantial economic losses. Bioassays confirmed that the So-PR strain exhibited a 141-fold higher resistance to phosphine than the So-PS strain (Table 1). In contrast, individual MBe treatment reversed this pattern, with the So-PR strain showing greater susceptibility, as indicated by its lower LC_50_, than the So-PS strain (Figure 3B, Table 1). When phosphine and MBe were applied in combination, strong insecticidal synergy was observed across a broad range of concentration combinations. Notably, supplementing phosphine with a sublethal concentration of MBe (LC_30_) resulted in 100% mortality in the So-PR strain (Figure 4 and Figure 5). These findings suggest that MBe is a promising biorational fumigant for managing phosphine-resistant pest populations, both as a stand-alone treatment and as a synergistic co-fumigant.

Molecular diagnostic analysis confirmed that phosphine resistance in So-PR was associated with an amino acid substitution from asparagine to threonine resulting from a point mutation in the *dld* gene (Figure 2). Point mutations in *dld* have been established as genetic determinants of phosphine resistance that enable insects to withstand phosphine-induced disruption of the mitochondrial respiratory chain [24]. Despite carrying this resistance-associated mutation, the So-PR strain showed no cross-resistance to MBe; instead, it exhibited greater sensitivity to MBe than the So-PS strain. This finding contrasts with previous observations in *Rhyzopertha dominica* (Coleoptera: Bostrichidae), in which MBe toxicity did not differ significantly between susceptible and resistant strains [25]. This difference may reflect the distinct physiological modes of action of the two fumigants: phosphine primarily disrupts mitochondrial respiration, whereas MBe has been reported to affect the central nervous system by inhibiting AChE activity [22]. Behaviorally, MBe-exposed *S. oryzae* exhibited characteristic knockdown symptoms, including supine positioning and involuntary leg tremors. These symptoms are consistent with AChE inhibition, which causes acetylcholine to accumulate at neuronal synapses, resulting in excessive neuroexcitation and muscle spasms [21]. The greater sensitivity of So-PR to MBe may represent negative cross-resistance, whereby physiological changes associated with phosphine resistance increase susceptibility to a different class of toxicant [31,32]. Consequently, even when pest populations develop resistance mechanisms against phosphine, they may remain susceptible to agents that target the nervous system through different pathways, such as those involving AChE inhibition [33,34]. Similar vulnerabilities to benzoate derivatives have been reported and patented for broader commercial applications against stored-product pests. Furthermore, the strong fumigation toxicity of MBe against phosphine-resistant strains has been reported across multiple stored-product pest species, supporting its potential as a resistance-management tool [35]. Overall, these findings suggest that MBe may exploit physiological differences associated with phosphine resistance to enhance control efficacy.

The Bliss independence analysis demonstrated strong synergy between the two fumigants, suggesting that their combined efficacy may result from simultaneous effects on distinct physiological targets (Figure 5). Detoxification processes under chemical stress are highly energy intensive and require substantial ATP consumption [36,37]. Phosphine disrupts mitochondrial electron transport, thereby impairing ATP production and promoting the accumulation of reactive oxygen species (ROS) [38,39]. Concurrently, MBe inhibits AChE, potentially inducing persistent neuroexcitation and muscle activity that further increases cellular energy demand [40,41]. Collectively, these effects suggest an alteration of cellular energy homeostasis, with MBe-induced neurotoxic stress occurring simultaneously with phosphine-mediated impairment of mitochondrial ATP production. This combined metabolic burden may contribute to the high mortality observed even at substantially reduced phosphine concentrations.

These findings have practical implications for stored-product protection and commercial quarantine operations. Due to its relatively low vapor pressure (0.38 mmHg at 25 °C) and moderate volatility, standalone liquid MBe may exhibit delayed action, often requiring extended exposure periods (>48 h) to achieve complete insecticidal efficacy [16]. However, in commercial quarantine settings, a strict 24-h operational window is required to prevent logistical bottlenecks and maintain commodity throughput. Importantly, our results demonstrate that MBe–phosphine co-fumigation overcomes these physical volatility limitations, driving strong synergistic mortality in phosphine-resistant *S. oryzae* within 24 h (Figure 4 and Figure 5). This rapid joint action confirms that co-fumigation can satisfy both commercial time constraints and high pest control standards.

Because female *S. oryzae* oviposit inside grain kernels where larvae develop internally [3,4], effective control requires fumigants capable of penetrating grain bulk [39]. In our laboratory bioassays, hermetic sealing and forced air circulation via magnetic stirring ensured uniform fumigant distribution during baseline screening (Figure 1C). However, in practical grain storage, gas distribution and overall efficacy are heavily influenced by grain mass, sorption, and penetration resistance. To address these practical constraints, further studies utilizing mini-silo systems with closed-loop circulation across incrementally increased grain volumes are warranted to rigorously evaluate sorption dynamics, gas penetration, and operational feasibility under realistic storage conditions.

For commercial application, however, appropriate gas-circulation systems will need to be evaluated to ensure homogeneous fumigant distribution throughout large storage silos. Furthermore, MBe has several characteristics favorable for use as a fumigant, including relatively low mammalian toxicity and approval as a food additive [19,42,43,44]. By reducing the amount of phosphine required for effective control, co-fumigation may also reduce phosphine exposure and potentially slow the selection of additional resistance.

In conclusion, these results indicate that MBe–phosphine co-fumigation represents a promising strategy for managing phosphine-resistant stored-product pests. Further field-scale trials in commercial grain silos are required to evaluate fumigant distribution, sorption, residue, efficacy against immature stages, and potential effects on grain quality parameters such as germination rate and end-use quality.

## Figures and Tables

**Table 1:** Lethal concentration values of phosphine and methyl benzoate for the PS and PR strains of *S. oryzae.*

Species	Chemical	Strains	LC_30_ (mg/L) (95% CI)	LC_50_ (mg/L) (95% CI)	LC_90_ (mg/L) (95% CI)	LC_99_ (mg/L) (95% CI)	Slope (±SE)	X^2^ (df)	*p* Value
*Sitophilus oryzae*	Phosphine	So-PS	0.006 (0.006–0.006)	0.008 (0.007–0.008)	0.014 (0.012–0.015)	0.022 (0.018–0.026)	5.21 ± 0.43	14.28 (23)	0.918
		So-PR	0.85 (0.78–0.91)	1.13 (1.06–1.20)	2.26 (2.03–2.62)	3.98 (3.32–5.10)	4.24 ± 0.34	41.05 (28)	0.053
	Methyl benzoate	So-PS	9.09 (8.84–9.31)	10.08 (9.86–10.31)	12.99 (12.57–13.53)	15.98 (15.17–17.05)	11.63 ± 0.70	34.11 (38)	0.650
		So-PR	5.14 (4.98–5.29)	6.10 (5.94–6.25)	9.24 (8.93–9.60)	12.98 (12.30–13.81)	7.09 ± 0.28	77.54 (77)	0.780

So-PS = phosphine-susceptible strain; So-PR = phosphine-resistant strain; CI = confidence interval; df = degrees of freedom.

## Data Availability

The original contributions presented in this study are included in the article. Further inquiries can be directed to the corresponding author.
